# WASH-nutrition integration as a global policy priority for antimicrobial stewardship in low- and middle-income countries

**DOI:** 10.1128/aem.02533-25

**Published:** 2026-02-17

**Authors:** O. J. Ikiba

**Affiliations:** 1 Microbiology Unit, Bayelsa State College of Nursing Sciences, Yenagoa, Bayelsa, Nigeria; Michigan State University, East Lansing, Michigan, USA

**Keywords:** antimicrobial resistance, antimicrobial stewardship, antibiotic doses averted

## Abstract

Antimicrobial resistance (AMR) is a global health threat predicted to hit 10 million deaths per year by 2050. Most conventional antimicrobial stewardship (AMS) strategies focus on clinical settings, failing to address community-level overuse, a primary driver of AMR in low- and middle-income countries. This commentary leverages novel evidence to argue for the prioritization of water, sanitation, and hygiene (WASH)-nutrition integration as a global health policy and first-line defense strategy against AMR. This argument is grounded on a target article that utilized causal mediation analysis to establish how low-cost household level WASH and nutrition interventions reduced pediatric antibiotic use via multiple biological pathways. This transforms prevention into a measurable antimicrobial defense strategy through antibiotic doses averted and limits antibiotic demand. These findings provide an empirical basis for integrated preventive measures, a quantifiable scorecard for securing political and fiscal commitment and redefinition of AMS as an element of global health policy and environmental conservation.

## COMMENTARY

Antibiotic overuse fuels antimicrobial resistance (AMR), a global health threat predicted to reach 10 million deaths per year worldwide by 2050 (1–3). Antimicrobial stewardship is a healthcare system-wide approach that monitors the judicious use of antimicrobials to guarantee their effectiveness and is vital in combating AMR (4). Strategies like prescriber education, restrictions on formulary, antibiotic cycling, and decision support systems have been proposed as measures to improve antibiotic use (5, 6). While these clinical strategies aim to regulate provider behavior, they are often ineffective in low- and middle-income countries (LMICs) where formal healthcare systems are bypassed (7). The integration of water, sanitation, and hygiene (WASH) and nutrition provides a biological counterbalance by targeting the environmental reservoirs of enteric pathogens that drive the need for initial treatment. This is relevant because, in contrast to high-income countries, many low- and middle-income countries (LMICs) face a high burden of infectious diseases, less strict regulations, and a high level of self-medication, which corresponds to abuse of antibiotics (2, 7).

Novel evidence from Nguyen et al. (8) proves that a WASH-nutrition integration was effective in reducing antibiotic use in children. Tested in a rural Bangladeshi village, the researchers treated water, upgraded latrines, installed handwashing stations, promoted maternal and infant nutrition practices, and provided lipid-based nutritional supplements to children aged 6 to 24 months. This led to a decline in caregiver-reported pediatric antibiotic use by 5.5 percentage points, while preventing enteric and respiratory infections (8). This reduction corresponds to roughly 1 antibiotic course avoided for every 18 children per year, indicating a significant decrease in the annual antibiotic burden. Such interventions directly lower the volume of antimicrobials entering the local ecosystem and exerting selection pressure on the normal microbiota. The critical breakthrough in this intervention is the establishment of antibiotic doses averted as a strong metric for antibiotic stewardship. Whereas traditional methods rely on restricting prescriptions to clinical settings (4–7), this model leverages WASH and nutrition interventions to control antibiotic demand and maintain population health (8).

## THE CAUSAL MECHANISM OF PREVENTION

In the study under review, the authors utilized causal mediation analysis (CMA) to establish the link between the interventions and the effects. Mediation analysis is a statistical technique used to understand how an intermediate variable called the mediator helps to explain how or why an independent variable influences an outcome (9). Traditional mediation methods are limited to binary outcomes that confirm associations but cannot establish causality. In contrast, causal mediation analysis works beyond linear models and can identify confounding assumptions and handle complex interactions between mediators (10, 11). Thus, the researchers were able to apply CMA to reinforce the causal interpretation that low-cost, household-level WASH and nutrition interventions reduced antibiotic use via multiple biological pathways. CMA also enabled the authors to investigate whether the drop in pediatric antibiotic use was mediated by reduced enteric virus carriage, diarrhea, or acute respiratory infections. By identifying that 38% of the total effect was mediated through these specific pathways, the study proves that reducing community pathogen load, especially triggers for inappropriate antibiotic use, prevents the enrichment of antibiotic resistance genes (ARGs) within the child’s gut microbiota. The randomized nature of the study helped the assumption that there was no unmeasured confounding of the treatment-outcome relationship. In sum, the use of CMA established a clear causal chain, one that pinpoints the specific mechanisms by which environmental modifications reduce antimicrobial selection pressure (8). The strength in this methodology is that it transforms WASH-nutrition from a conventional public health concept into a measurable antimicrobial defense strategy. This evidence reiterates the fact that eliminating the risk of infection is a sustainable approach for population-level drug conservation, a clear pathway to reducing dependence on antibiotics. Table 1 summarizes the causal mediation effect.

**TABLE 1:** Causal pathways linking WASH and nutrition interventions to reduced caregiver-reported antibiotic use^*a*^

Component	Description/mediator status	Intervention → mediator (X → M)	Mediator → outcome (M → Y)	NIE contribution (percentage points)
Intervention (X)	WASH + nutrition package			
Outcome (Y)	Reduced caregiver-reported antibiotic use among children			
Total effect (X → Y)	Overall reduction in antibiotic use			–5.5 pp (95% CI: 1.2, 9.9)
Natural direct effect (NDE)	Effect not mediated by the measured infection pathways			–3.4 pp (62% of total effect)
Natural indirect effect (NIE)	Effect mediated through the measured infection pathways			–2.1 pp (38% of total effect)
Mediator: enteric virus carriage	Infection reduction pathway	PR 0.60 (95% CI: 0.50, 0.71)	Associated with 15% higher antibiotic use (PR 1.15)	–1.5 pp
Mediator: ARI with fever	Infection reduction pathway	Reduced prevalence by 26% (95% CI: 9%, 40%)	Associated with 40%–50% higher antibiotic use	–0.7 pp
Mediator: diarrhea	Infection reduction pathway	Reduced prevalence by 33% (95% CI: 11%, 49%)	Associated with 27% higher antibiotic use (PR 1.27)	–0.6 pp

^*a*^ The total effect equals the natural direct effect and natural indirect effect. The NIE is the combined contribution of mediator pathways. The findings showed that the combined intervention package reduced pediatric antibiotic use, with nearly 38% of the effect due to reductions in enteric and respiratory infections. ARI, acute respiratory infection; pp, percentage points; NIE, natural indirect effect; NDE, natural direct effect; PR, prevalence ratio; CI, confidence interval.

## CHANGING THE PARADIGM OF ANTIMICROBIAL STEWARDSHIP

The findings from Nguyen et al. (8) provide empirical evidence to argue for a policy shift in public health intervention. The study demonstrated that a WASH-nutrition integration was potent in reducing pediatric antibiotic use. This implies that these low-cost household level interventions should take priority as a first-line defense strategy against AMR. There should be a shift both in funding and implementation from their traditional categorization as development aid. The urgency for this shift is obvious; the magnitude and threat of AMR were linked to nearly 4.95 million deaths worldwide in 2019 (12). Moreover, high rates of antibiotic exposure are common in young children in these settings (13, 14). By intervening to prevent these infections, drug exposure in young children becomes limited while conserving global antibiotic efficacy.

## CONCLUSION

The identification of the mechanisms by which low-cost household level WASH and nutrition interventions reduced pediatric antibiotic use provides a concrete blueprint for upstream integration of environmental health into antimicrobial stewardship in line with One Health principles. This approach targets the ecological root of AMR by reducing enteric pathogen reservoirs and moderating the propagation of ARGs within highly susceptible populations, such as children aged 14–28 months. Future research should address the long-term ecological impact of these interventions by including antibiotic resistance carriage as an outcome. Genomic surveillance methods should be used to track reductions in antibiotic resistance genes (ARGs) in community settings per evidence that improved WASH can reduce ARGs. In sum, stewardship must be understood not merely as a clinical practice control but as an act of fundamental environmental conservation.
